# Association between Potentially Inappropriate Medication Use and Chronic Diseases in the Elderly

**DOI:** 10.3390/ijerph16122189

**Published:** 2019-06-20

**Authors:** Tzu-Chueh Wang, Pou-Jen Ku, Hai-Lin Lu, Kung-Chuan Hsu, Damien Trezise, Hue-Yu Wang

**Affiliations:** 1 Department of Pharmacy, Chia Nan University of Pharmacy and Science, Tainan City 71710, Taiwan; tzchwa@mail.cnu.edu.tw; 2 Taiwan Pharmacist Association, Taipei City 10452, Taiwan; kupoujen@yahoo.com.tw; 3 Department of Information Management, Chia Nan University of Pharmacy and Science, Tainan City 71710, Taiwan; hllu2719@gmail.com; 4 Giraffe Pharmacy, Tainan City 71049, Taiwan; gighv206@gmail.com; 5 Department of Applied Foreign Languages, Chia Nan University of Pharmacy and Science, Tainan City 71710, Taiwan; damien@mail.cnu.edu.tw; 6 Department of Pharmacy, Chi-Mei Medical Center, Tainan City 71004, Taiwan; cmh5500wang@gmail.com

**Keywords:** Beers Criteria, chronic disease, elderly, Potentially Inappropriate Medication

## Abstract

Long-term continuous exposure to potentially inappropriate medications (PIMs) can lead to adverse events in the elderly. However, the effects of long-term exposure of the elderly to PIM and the relationship between PIM and chronic diseases remain unclear. The objective of this study was to investigate the continuous use of PIMs in a community-dwelling elderly population. A cross-sectional population-based study was conducted using community pharmacy–filed dispensing records from the Hcare system. Twenty-three community pharmacies were sampled from 2013 to 2015 to obtain records of patients above 65 years-old with continuous prescriptions. PIM were identified according to the 2015 Beers Criteria. The prevalence of patients using PIM was highest in patients with co-morbid mental disorders (40.05%), followed by neurological system disorders (28.91%). Patients who were prescribed a PIM were more than three times as likely to have a mental disorder as those (odds ratio 3.16, 95% confidence interval: 3.06–3.28) with non-chronic diseases. The most prescribed PIM agents were central nervous system drugs (53.16%), and benzodiazepines (35.15%). Patients with mental disorders had the highest rate of long-term persistent PIM exposure, with benzodiazepines being the most frequently dispensed. Drug safety concerns should be closely monitored in elderly patients with the abovementioned conditions.

## 1. Introduction

Studies show that the use of pharmaceuticals in developed countries increases with age and that elderly people often take at least three prescription drugs simultaneously [1,2]. Even in developing countries, the proportion of elderly who take at least one drug daily is as high as 85–90% [3,4,5]. Because of the varying physiological, pharmacokinetic and pharmacodynamic characteristics of the elderly, it has become difficult for physicians to prescribe drugs. This problem can result in frequent prescriptions of potentially inappropriate medications (PIMs). The higher the number of drugs taken by an elderly patient, the greater the likelihood of the patient being subjected to potentially inappropriate drug therapy, consequently increasing the risk of drug-related adverse reactions [6,7]. The current rate of PIM prescription to the elderly is an important public health issue. Not only does it affect patients’ morbidity, hospitalization and mortality rates, but adverse events from PIMs add significantly to healthcare cost [8,9].

The Beers Criteria constitute a consensus-based list of PIMs for individuals aged 65 years and older. They were first developed for nursing home residents in 1991 and have been updated twice, in 1997 and 2003, for use in the general elderly population based on new clinical evidence [10]. In 2015, the list of select drugs that should be avoided or subjected to dosage adjustments according to patient’s kidney function or select drug–drug interactions harmful to older adults was updated [11]. A new version was released in 2019, and differences between this and the 2015 version will be described in the discussion section. Studies on physicians prescribing PIM to the elderly and use of the Beers Criteria have been published worldwide, including cost [12,13], risk factors [14,15], prevalence, and PIM-related effects [9,16,17]. 

Taiwan’s National Health Insurance (NHI) system is a specialist treatment system, in which insured persons have unrestricted choices of hospitals and unlimited numbers of visits. From the first consultation, doctors can issue prescriptions for chronic diseases that allow patients to utilize community pharmacies for three consecutive months without having to return for a follow-up consultation. This practice exposes the elderly to an increased risk of PIM. Previous studies have investigated the prevalence of PIM amongst the elderly [16], risk of adverse reactions caused by PIM in chronic disease treatment [18], safety of single-category PIMs (anti-cholinergics) used by elderly patients and dangers of PIM use in elderly patients in continuous care [19,20]. The results highlight the seriousness of this issue, showing that PIM prescriptions are associated with potential risks to the elderly. The proportion of elderly people with at least one chronic disease is about 50% [21,22,23], and they generally require long-term use of medications to manage symptoms. This raises two questions worthy of further investigation: (1) What proportion of elderly people is subject to long-term exposure to PIM? (2) What is the relationship between PIM prescription and chronic diseases already diagnosed?

The claims data including prescriptions filed by authorized community pharmacies were used to investigate the risk of long-term continuous (three-year period) exposure to PIM amongst patients aged 65 years or older with chronic diseases. This study aimed to investigate the relationships between PIM and chronic diseases and the distribution of PIMs.

## 2. Materials and Methods

### 2.1. Data Sources and Study Design

This was a population-based study using community pharmacy-filed dispensing records in the Community Pharmacy Care Management System (Hcare) of the Taiwan Pharmacists Association from January 1, 2013, to December 31, 2015. Population data were drawn from a random sample of eligible community pharmacies that met the research criteria.

The initial selection conditions included pharmacies that filled an average of more than 900 prescriptions per month. Pharmacies in which an undue proportion of the prescriptions filled came from a single medical institution, such as more than 50% of total prescriptions being issued by one hospital, were excluded to reduce sampling bias.

A total of 23 community pharmacies met the selection criteria and were included in the study. Information in claims data files was obtained after removing identifying personal information, and included monthly claims data of the community pharmacy, age and sex of the insured, hospital and department that issued the prescription, patient’s diagnosis (classified according to the International Classification of Diseases, 9th, ICD-9), medication dispensed (including the generic name, dose, administration method, number of days of prescription and date of issue) and service center. We included data for oral medications and insulin. This study used the 2015 Beers Criteria to determine whether medicines prescribed by doctors were PIMs or not. 

### 2.2. Study Population

**Figure 1:**
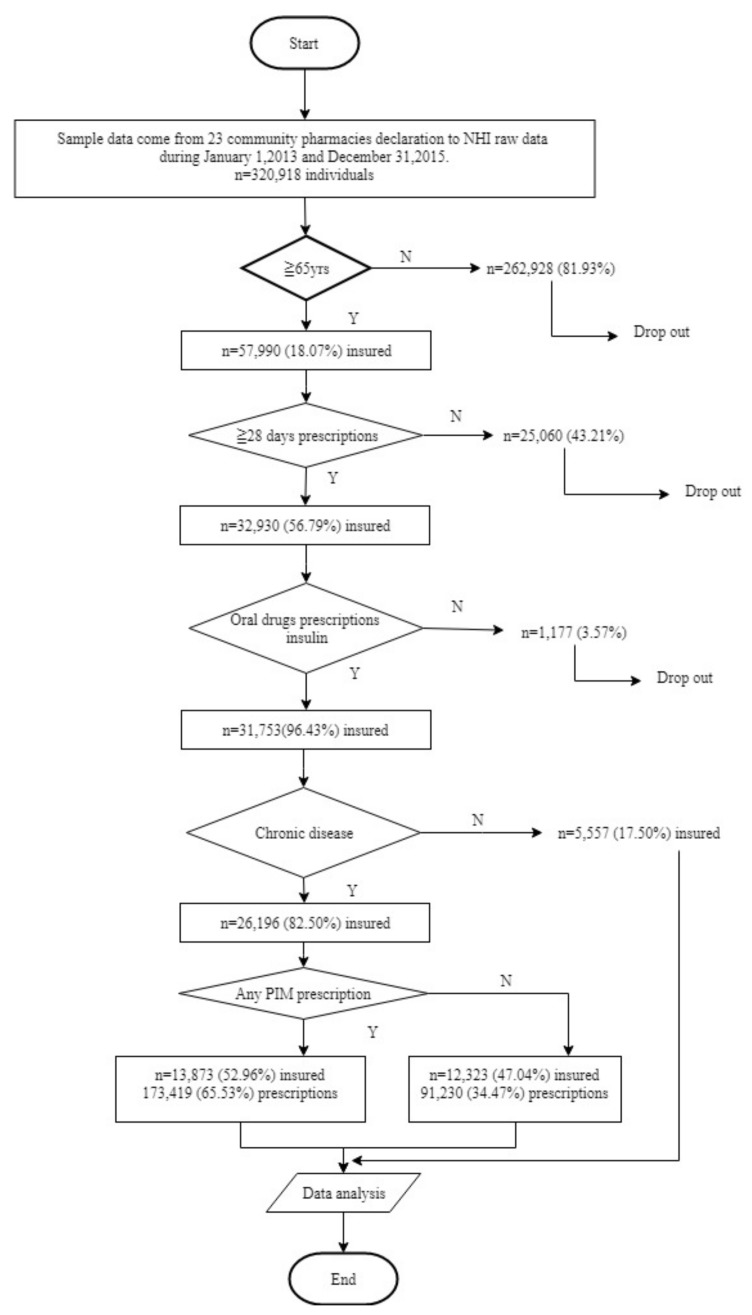
Flowchart of patient sample collection.

The research subjects were Taiwanese people aged 65 years or older who were insured under the Taiwan NHI and had received more than 28 consecutive days of prescriptions for chronic disease at an NHI-approved pharmacy. Subjects whose main diagnosis was a chronic disease were further classified into PIM and non-PIM groups after analysis of their prescriptions. Patients with non-chronic conditions that met the same criteria comprised the control group. The selection process according to demographic data is shown in Figure 1.

### 2.3. Definitions of Terms

Chronic diseases: Those that persist for more than three months or cause permanent injuries or conditions that persist due to illness or congenital complaints [24]. Chronic diseases in this study were classified according to the 16 major chronic disease categories recognized by the Taiwan Health Insurance Department [25].

Prescriptions: Prescriptions written by doctors that are valid for up to 30 days in Taiwan, and can only be filled once. Continuous prescriptions are available for patients with chronic diseases. 

Continuous prescriptions for chronic diseases: Prescriptions issued by physicians to patients diagnosed with chronic diseases that can be refilled up to three times, for up to 30 days each time.

National Health Insurance (NHI)–approved pharmacy: A community pharmacy that accepts continuous prescriptions for chronic diseases prescribed by hospitals or clinics.

Potentially inappropriate medication (PIM): Medications designated as potentially inappropriate for use in older adults according to the American Geriatrics Society’s 2015 updated Beers Criteria (adults) [11].

Patients with PIM prescriptions: Any person who was prescribed at least one medication designated as a PIM during the study period.

Patients without PIM prescriptions: Any person who was never prescribed a PIM, as defined above.

### 2.4. Measurements

Subject data were compiled under fields including age, sex, prescription source, prescribing department, illness diagnosed and names of prescribed medications. The probability of prescriptions for chronic diseases causing patients to experience long-term exposure to PIM during the study period was designated as individual exposure to PIM (IE_IPIM_). The formula for the IE_IPM_ calculation is shown below as Formula (1):

IE_IPIM_ = (number of PIM prescribed to an individual patient I_PIM_÷total number of medicines prescribed for that patient I_Total_) X100%.

The incidence of IEIPIM in patients with chronic diseases exposed to different rates of PIM for extended periods was designated as RPIM (rate of PIM). The formula for calculating RPIM is shown below as Formula (2), and the number of PIM prescriptions for chronic diseases is as follows:

PIM I_CDpim_÷total number of prescriptions for chronic diseases I_CDTotal_X100%.

Using the odds ratio (OR), a comparison of the risk of PIM being prescribed to patients with any chronic disease and those with non-chronic diseases could be made under equivalent conditions. Finally, the most commonly prescribed PIMs were analyzed in terms of their distribution according to the 2015 Beers Criteria.

(1) $$IE_{IPIM}=\frac{I_{PIM}}{I_{Total}}\times 100\%$$

(2) $$R_{PIM}=\frac{I_{CDpim}}{I_{CDTotal}}\times 100\%$$

### 2.5. Statistical Analysis

Chi-square tests were used to determine the independence of categorical variables. Multivariate logistic regression analysis was performed to evaluate the association between each chronic disease and PIM prescriptions. The results are reported as ORs and 95% confidence intervals (CI), after adjusting for potential confounding factors such as age, institutional level and medical department. Statistical significance was set at p < 0.05. All analyses were conducted using STATA software, version 14.2 (STATA Corp., College Station, TX, USA).

### 2.6. Ethics Approval

This study was approved by the Institutional Review Board of Chi-Mei Medical Center (IRB serial no. 10706-003). Data was converted from the Hcare system and hence the patients’ personal information was deleted.

## 3. Results

### 3.1. Subjects’ Characteristics

The process for identifying the data for this research is shown in Figure 1. A total of 320,918 insured persons’ data files were retrieved from 23 Hcare community pharmacies, from which a total of 13,873 insured persons met the inclusion criteria (elderly persons with chronic diseases who had been prescribed PIM). A total of 173,419 PIM prescriptions were identified (65.53%).

Table 1 shows that the incidence of PIM prescriptions amongst elderly patients with chronic diseases increased with age and this correlation was significant (*p* < 0.001).

There was a significant positive correlation between the number of co-morbidities and the incidence of PIM prescriptions (*p* < 0.001). However, there was no significant correlation between sex and PIM prescriptions (*p* = 0.576). There was a correlation between polypharmacy prescriptions and the incidence of PIM, in that the incidence of PIM was lower when less than five medications were taken (approximately 31.98% vs. 57.66%).

### 3.2. Probability of Sustained Exposure to PIM Amongst Patients with Various Chronic Diseases

**Figure 2:**
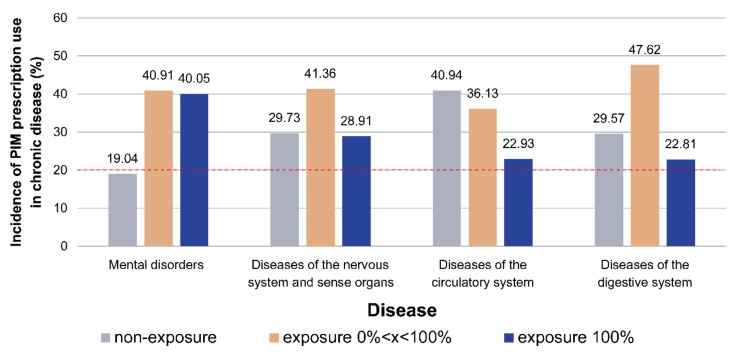
Chronic diseases for which there was a 100% continuous long-term exposure to PIM prescription rate of >20%, shown at exposure rates of 0%, 0% <x<100% and 100%.

Figure 2 shows in all chronic diseases, PIM was included in each prescription (100% exposure) for a significant number of cases, with the highest incidences in mental (40.05%), neurological (28.91%), circulatory (22.93%), and respiratory (22.81%) conditions. 

The distribution of the incidence of long-term continuous exposure to PIM of >20% for four types of chronic diseases (0%, 0% < x <100%, 100%) is shown in Figure 2. Patients with chronic diseases of the circulatory system had the highest probability of zero exposure to PIM (41%), whilst the highest probability of 100% exposure to PIM occurred amongst patients with mental disorders (19.04%). 

Table 2 shows that the top three chronic diseases in terms of the total number of prescriptions issued were endocrine and metabolic (1,218,545), circulatory system (109,119) and musculoskeletal system and connective tissue diseases (45,063). However, the top three chronic diseases in terms of the incidence of PIM prescriptions were mental (55.85%), neurological (47.92%) and genitourinary system diseases (43.4%).

When multivariate logistic regression analysis was used to compare the ORs of PIM prescriptions for chronic and non-chronic diseases, the ORs of PIM prescriptions for mental disorders was 3.16 times that of PIM prescriptions for non-chronic diseases (95% CI, 3.06–3.28) and 1.72 times higher that of PIM prescriptions for chronic neurological diseases, which was the next highest category (95% CI, 1.64–1.80).

Compared with patients ≧65 years with oral drug prescriptions only and whose main diagnosis did not meet the criteria for chronic diseases.

### 3.3. Analysis of Drugs Prescribed to Patients with Chronic Diseases and Long-term Continuous Exposure to PIM Prescriptions

Table 3 shows the distribution of drug categories for long-term continuous exposure to PIM in the top four chronic diseases. The most prescribed were the category of central nervous system drugs, accounting for 53.16% (68,425) of all drugs. Of these, the highest number of prescriptions was for benzodiazepines, which are the most frequently issued drugs for nervous system diseases and mental disorders. The second most frequently prescribed category of drugs was for the treatment of cardiovascular disorders, comprising 20.32% of the total (26,157), and alpha-1 blockers were the most prescribed.

## 4. Discussion

### 4.1. Subject Characteristics

Previous studies on PIM prescriptions to the elderly showed that sex, age, co-morbidity and polypharmacy were significant factors affecting the prescribed patients [17,18,26,27,28]. Those results are similar to the results of the present study (Table 1). Although the correlation between sex and the incidence of PIM did not reach statistical significance (*p* = 0.576), the percentage of PIM prescribed to women was higher than the percentage prescribed to men (54.55% vs. 45.45%).

The fact that cardiology departments issued PIM prescriptions to the highest number of patients (3,523) in this study was in line with the findings of a 2015 study on the prevalence of PIM prescribed to hospitalized patients older than 65 years [29]. However, the highest incidence occurred in psychiatric departments (88.43%). The results are similar to those of Lang et al., who reported that the PIM prevalence rate in psychiatric inpatients or outpatients was as high as 53.0% [30]. 

### 4.2. Long-term Exposure to PIM Prescriptions Amongst Patients with Various Chronic Diseases

People age 65 years and older, with at least one chronic disease are usually multiple medication users [9,10,17,18,31,32,33]. The proportion who take at least one PIM is between 22 and 56% [34,35]. Thus, the elderly have long been in serious danger of exposure to PIM. Our study went a step further and explored the long-term risks of PIM exposure amongst the community-dwelling elderly with chronic diseases. Our study shows that 52.96% of chronically ill elderly people in the communities were taking PIM; patients with chronic mental disorders were the most likely to have at least one PIM on every prescription (40.05%). This shows that out of all chronic diseases, patients with mental disorders are at the greatest risk of being prescribed PIM. Compared to patients with non-chronic diseases, the OR for being prescribed PIM due to mental disorders was at least double that of any other disease (Table 2). This finding is worthy of clinicians’ attention. 

Many studies proved high correlations between chronic mental disorders and PIM prescriptions, which is in exact accordance with the findings of this study. Yang et al. studied the relationship between elderly patients with chronic diseases or disabilities and PIM prescriptions. The results showed a significant correlation between prescriptions for PIM and the presence of a mental disorder (*p* < 0.001) [33]. Vieira de Lima et al. also showed that a mental disorder diagnosis was one of the factors that contributed to the high rate of PIM prescriptions amongst the elderly in institutions [36]. Swanoski et al. found that the incidence of PIM prescriptions amongst patients with diabetes was higher than that for depression, which differs from the findings of this and other studies [28]. This may result from their investigation of only three chronic diseases—diabetes, arthritis and depression. Compared to the broad number of chronic diseases reviewed in the current study, the investigation of a different sample group inevitably produced different results. The incidence of long-term continuous exposure to PIM prescriptions was second-highest in neurological diseases. This is in accordance with past studies by Tsai et al. that identified neurological patients with cognitive impairments as having a higher risk of exposure to PIM to hence the general population [37].

### 4.3. Correlation between Different Chronic Diseases and PIM

As shown in Table 3, prescriptions for CNS category medications accounted for more than half (53.16%) of all drug categories, and benzodiazepines were the most commonly prescribed, with 45,242 records, constituting about 35.15% of the total. This finding is similar to those of past research [17,38,39]. These drugs are commonly used to treat anxiety, psychological distress, and/or insomnia in the elderly. Age-related changes in the elderly can also influence an individual’s pharmacokinetics and pharmacodynamics [39]. This is the main reason why the elderly are prone to unexpected adverse effects after taking these drugs. There is also a high incidence of adverse drug events (ADEs) caused by anti-psychotic drug use amongst outpatients [40]. In particular, drugs with inhibitory effects on the CNS often lead to sedation, extrapyramidal symptoms, dizziness and ataxia and account for more than 55% of ADEs. In addition, most anti-psychotic drugs are metabolized by the hepatic cytochrome P450 system, and patients who are taking drugs that compete metabolically with anti-psychotics can experience potentially harmful drug interactions [41]. Hence, the aforementioned commonly prescribed mental disorder drugs present high potential risk to the elderly.

Cardiovascular drugs were the second most frequent PIM in this study. The most commonly prescribed were alpha-1 blockers, such as doxazosin, terazosin and tamsulosin. These are recommended agents for treating hypertension with benign prostate hypertrophy. However, they carry potential ADEs such as postural hypotension and dizziness; hence, they are not recommended in elderly patients on routine hypertension treatment. If these medications are necessary for the treatment of prostatic hypertrophy, patients should be advised to take them before going to bed. Otherwise, alternative medications are recommended whenever possible. Metoclopramide was the most commonly prescribed drug for digestive tract diseases. The potential risk of tardive dyskinesia for elderly using metoclopramide is common [42]; thus, it is suggested that metoclopramide be replaced with other drugs if necessary. 

### 4.4. Differences between Beers Criteria 2019 and 2015

The American Geriatrics Society updated the Beers Criteria in 2019, removing Ticlopidine and Pentazocine because they were no longer on the US market, and adding Glimepiride, Methylscopolamine and Pyrilamine [43]. When the Beers Criteria were updated in 2015, they could be immediately applied to the Taiwan environment, because there were no substantial differences in the pharmaceuticals available on the market. However, Ticlopidine is still widely used in Taiwan. Therefore, use of the 2019 version of the Beers Criteria 2019 to determine the rationality of prescriptions in the Taiwan context may have resulted in Ticlopidine being overlooked, and exposed elderly patients to unsafe antithrombotic drugs.

### 4.5. Limitations of the Study

This study was aimed at evaluating community-dwelling elderly with chronic diseases, and the study population was not drawn from all areas of Taiwan. However, we tried to avoid an over-concentration of drug prescription data from any one kind of medical institution and ensured that the prescriptions originated from all healthcare organization levels, including hospitals and community clinics, to obtain more objective data. Therefore, we believe that the results of this study may represent the current state of PIM use amongst elderly patients with chronic diseases in Taiwan. In subsequent studies, we will further address and evaluate the clinical outcomes and relative risks amongst patients taking PIMs.

## 5. Conclusions

The probability of PIM prescriptions in the elderly with chronic diseases was significantly correlated with age, sex, co-morbidity and polypharmacy. The mental disorders category had the highest rate of long-term persistent exposure to PIM. The most common prescriptions for PIM were in the CNS category, with benzodiazepines being the most frequently dispensed. Drug safety concerns should be closely monitored in the elderly with the abovementioned conditions.

## Figures and Tables

**Table 1:** Study participants’ demographic and clinical characteristics.

Characteristic	Patients with PIM Prescriptions (*n* = 16,766)	Patients without PIM Prescriptions (*n* = 14,987)	*p*-Value *
Age			*<0.001* ***
65–74 years	8130(48.49)	8442(56.33)	*<0.001* ***
75–84 years	6242(37.23)	4889(32.62)	*<0.001* ***
≥85 years	2394(14.28)	1656(11.05)	reference
Sex			*0.096*
Male	7620(45.45)	6672(44.52)	
Female	9146(54.55)	8315(55.48)	
Department			*<0.001* ***
Cardiology	4629(15.6)	3248(16.97)	0.022 *
Chest	1032(3.48)	496(2.59)	*<0.001* ***
Endocrinology	1696(5.72)	1132(5.91)	0.361
Family medicine	3785(12.75)	3574(18.67)	*<0.001* ***
Gastroenterology	2040(6.88)	612(3.2)	*<0.001* ***
Internal medicine	3987(13.43)	3961(20.7)	*<0.001* ***
Nephrology	1053(3.55)	456(2.38)	*<0.001* ***
Neurology	3886(13.09)	1528(7.99)	*<0.001* ***
Neurosurgery	707(2.38)	368(1.92)	0.007 **
Orthopedics	1528(5.15)	1058(5.53)	0.116
Others	1945(6.55)	1237(6.47)	reference
Psychiatry	1236(4.16)	144(0.75)	*<0.001* ***
Surgery	500(1.68)	379(1.98)	0.023 *
Urology	1655(5.58)	945(4.94)	0.048 *
Number of chronic disease			*<0.001* ***
1	4281(25.53)	6495(43.34)	*<0.001* ***
2	5197(31)	4701(31.37)	*<0.001* ***
3	3585(21.38)	2619(17.48)	*<0.001* ***
4	2116(12.62)	862(5.75)	*<0.001* ***
≧5	1587(9.47)	310(2.07)	reference

Values are presented as *n* (%). *χ^2^ tests. * *p* < 0.05, ** *p* < 0.01, *** *p* < 0.001.

**Table 2:** Comparison of 100% continuous exposure to PIM prescriptions, number of prescriptions, PIM prescription rate and OR by chronic disease type.

Classification of Chronic Disease	Number of Patients with Chronic Diseases	Patients with 100% Continuous Exposure to PIM Prescriptions, n (%)	Number of Chronic Disease Prescriptions	PIM Prescription Rate, n (%)	PIM Prescription, OR^a^	95% CI
Neoplasms	1441	236 (16.38)	17,593	5983(34.01)	0.83 ***	0.79–0.87
Endocrine, nutritional and metabolic diseases and immunity disorders	8798	1304 (14.82)	121,854	41,141 (33.76)	0.82 ***	0.8–0.84
Mental disorders	3618	1449 (40.05)	48,316	26,986 (55.85)	3.16 ***	3.06–3.28
Diseases of the nervous system and sense organs	1463	423 (28.91)	19,850	9512 (47.92)	1.72 ***	1.64–1.80
Diseases of the circulatory system	8491	1947 (22.93)	109,119	43,761 (40.10)	1.33 ***	1.31–1.36
Diseases of the respiratory system	1774	252 (14.21)	24,664	9338 (37.86)	0.78 ***	0.74–0.82
Diseases of the digestive system	2012	459 (22.81)	27,846	11,446 (41.10)	1.09 **	1.04–1.15
Diseases of the genitourinary system	1201	226 (18.82)	17,105	7423 (43.40)	1.65 ***	1.56–1.74
Diseases of the musculoskeletal system and connective tissue	3793	579 (15.26)	45,063	16,784 (37.25)	1.05 **	1.01–1.10
Diseases of the eye and its accessory organs	182	7 (3.85)	2005	605 (30.17)	0.24 ***	0.18–0.32
Infectious and parasitic diseases	44	5 (11.36)	224	66 (29.46)	0.64 *	0.43–0.93
Congenital defects	131	22 (16.79)	1768	739 (41.80)	1.46 ***	1.24–1.72
Diseases of the skin and subcutaneous tissue	513	36 (7.02)	8177	2906 (35.54)	0.46 ***	0.41–0.52
Diseases of the blood and blood-forming organs	217	28 (12.90)	3180	1128 (35.47)	0.73 ***	0.63–0.85
Diseases of the ear and mastoid	415	72 (17.35)	6333	2318 (36.6)	1.05	0.95–1.15
Other	7505	1328 (17.69)	104,392	38,933 (37.3)	1.18 ***	1.15–1.21

CI: confidence interval; OR: odds ratio; PIMs: potentially inappropriate medications; * p < 0.05, ** p < 0.01, *** p < 0.001.

**Table 3:** Distribution of long-term continuous exposure to PIM in certain chronic diseases.

PIM	Total (%)	Mental Disorders	Diseases of the Nervous System	Diseases of the Circulatory System	Diseases of the Digestive System
Anti-cholinergics	6230 (4.84%)				
First-generation anti-histamines	3321	833(25.08%)	437(13.16%)	1579(47.55%)	472(14.21%)
Anti-Parkinsonian agents	2159	618(28.62%)	1006(46.6%)	449(20.8%)	86(3.98%)
Anti-spasmodics	750	179(23.87%)	70(9.33%)	299(39.87%)	202(26.93%)
Anti-thrombotics	3416 (2.65%)				
Ticlopidine	3416	436(12.76%)	221(6.47%)	2384(69.79%)	375(10.98%)
Cardiovascular	26,157 (20.32%)				
Peripheral alpha-1 blockers	8753	1346(15.38%)	641(7.32%)	5713(65.27%)	1053(12.03%)
Central alpha blockers	935	79(8.45%)	24(2.57%)	623(66.63%)	209(22.35%)
Dronedarone	3288	327(9.95%)	150(4.56%)	2638(80.23%)	173(5.26%)
Digoxin	3181	299(9.4%)	185(5.82%)	2470(77.65%)	227(7.14%)
Amiodarone	935	79(8.45%)	24(2.57%)	623(66.63%)	209(22.35%)
Central nervous system	68,425 (53.16%)				
Anti-depressants, alone or in combination	3501	1530(43.7%)	428(12.23%)	1189(33.96%)	354(10.11%)
Anti-psychotics, first- (conventional) and second- (atypical) generation	9087	4854(53.42%)	1223(13.46%)	2457(27.04%)	553(6.09%)
Benzodiazepines	45,242	17,977(39.74%)	5105(11.28%)	17,699(39.12%)	4461(9.86%)
Non-benzodiazepine, benzodiazepine receptor agonist hypnotics	10,588	5124(48.39%)	773(7.3%)	3673(34.69%)	1018(9.61%)
Endocrine	7094 (5.51%)				
Estrogens with or without progestins	327	92(28.13%)	39(11.93%)	168(51.38%)	28(8.56%)
Insulin, sliding scale	5389	1112(20.63%)	633(11.75%)	2857(53.02%)	787(14.6%)
Megestrol	5	2(40%)	0(0%)	2(40%)	1(20%)
Glyburide	1373	314(22.87%)	76(5.54%)	751(54.7%)	232(16.9%)
Gastrointestinal	8666 (6.73%)				
Metoclopramide	5961	1134(19.02%)	474(7.95%)	2428(40.73%)	1925(32.29%)
Proton-pump inhibitors	2705	294(10.87%)	106(3.92%)	863(31.9%)	1442(53.31%)
Pain medications	5435 (4.22%)				
Non-selective cyclooxygenase inhibitors	3300	809(24.52%)	488(14.79%)	1581(47.91%)	422(12.79%)
Indomethacin	217	39(17.97%)	12(5.53%)	110(50.69%)	56(25.81%)
Skeletal muscle relaxants	1918	502(26.17%)	285(14.86%)	824(42.96%)	307(16.01%)
Genitourinary	3300(2.56)				
Desmopressin	3300	173(5.24%)	73(2.21%)	264(8%)	133(4.03%)

PIM: potentially inappropriate medications according to 2015 Beers criteria.
